# Experimental methods for the Palaeolithic dry distillation of birch bark: implications for the origin and development of Neandertal adhesive technology

**DOI:** 10.1038/s41598-017-08106-7

**Published:** 2017-08-31

**Authors:** P. R. B. Kozowyk, M. Soressi, D. Pomstra, G. H. J. Langejans

**Affiliations:** 1 Faculty of Archaeology, Leiden University, Leiden, The Netherlands; 2 Het Stenen Tijdperk, Wezep, The Netherlands; 3 Centre for Anthropological Research & Department of Anthropology and Development Studies, University of Johannesburg, Johannesburg, South Africa

## Abstract

The destructive distillation of birch bark to produce tar has recently featured in debates about the technological and cognitive abilities of Neandertals and modern humans. The abilities to precisely control fire temperatures and to manipulate adhesive properties are believed to require advanced mental traits. However, the significance given to adhesive technology in these debates has quickly outgrown our understanding of birch bark tar and its manufacture using aceramic techniques. In this paper, we detail three experimental methods of Palaeolithic tar production ranging from simple to complex. We recorded the fuel, time, materials, temperatures, and tar yield for each method and compared them with the tar known from the Palaeolithic. Our results indicate that it is possible to obtain useful amounts of tar by combining materials and technology already in use by Neandertals. A ceramic container is not required, and temperature ﻿control﻿ need not be as precise as previously thought. However, Neandertals must have been able to recognize certain material properties, such as adhesive tack and viscosity. In this way, they could develop the technology from producing small traces of tar on partially burned bark to techniques capable of manufacturing quantities of tar equal to those found in the Middle Palaeolithi﻿c archaeological record.

## Introduction

The manufacture and use of adhesives for hafting has become a focal point in the debate about the cognitive and technological capabilities of Neandertals and early modern humans^1–7^. Adhesives are one of the earliest transformative technologies known^8^ and tar production is at least 200 thousand years old (ka)^9^. Tar is synthesized from the dry (destructive) distillation of organic material, commonly birch bark (*Betula* sp.) or pine wood (*Pinus* sp.). Tar distillation is thought to be a complicated process requiring forward planning, knowledge of materials and abstraction^7, 10^. The oldest known tar-hafted stone tools were discovered at a Middle Pleistocene site in Italy, during a time when only Neandertals were present in Europe^9^. Tar lumps and adhesive residues on stone tools were also found at two Neandertal sites in Germany dating to 40–80 ka and ~120 ka respectively^10, 11^. Direct evidence for adhesive use in Africa is more numerous but only goes back to ~70 ka^2, 12^. It has been argued that the innovative nature of compound adhesive manufacture in southern Africa is a proxy for complex cognition^4, 13^. Yet compound adhesives share many similarities to birch bark tar production^7, 10^ and may be equally sensitive to additives or post-production processes^14^. Tar production in Palaeolithic Europe has in turn been used to argue for similarities between the technological capabilities of Neandertals and their near-modern contemporaries in Africa^3, 7, 15^. It is presently unknown why evidence of tar production by modern humans is much younger, but if birch bark is more suitable for making tar than other materials, then the absence of birch in Africa might be one explanation.

In historic and modern periods, tar was produced on an industrial scale using large earth mounds, or in kilns using ceramic or metallic containers. It is unclear how tar was produced during the Pleistocene when ceramic containers are rare or unknown. Previous experimental attempts at tar manufacture using aceramic or Palaeolithic technology often lack detail. Furthermore the resulting tar yield is unknown or too small to be measured (e.g. superficial residues coating a thermocouple^16^), and are thus not enough to effectively haft a tool^17–21^. The significance that birch tar production is given in debates about Neandertal and modern human technology and cognition (cf. refs 3, 7, 15 but see also ref. 22) has therefore outgrown our knowledge of the material and its production processes. We cannot fully understand the cognitive complexities and reconstruct the required degree of innovation associated with tar manufacture if we do not know what production methods were available.

Here we present an experimental study testing the dry distillation of birch bark to produce tar using variations of previously explored potential Palaeolithic techniques: the ‘ash mound’ (AM) method^19^, the ‘pit roll’ (PR) or cigar roll method^11, 23, 24^, and the ‘raised structure’ (RS) method^16–18, 20, 25, 26^. We assessed these production methods in three ways:

1. Yield – time and fuel spent versus tar quantity obtained,
2. Temperature – required degree of temperature control to successfully produce tar,
3. Complexity – number of individual components (cf. technounits^27^) and the number of steps^28^ required to produce tar.

The detailed account of aceramic tar production methods described here provide a new empirical baseline to reconstruct the origin and the evolution of tar technology and its associated cognitive skills through the Pleistocene.

## Results

### Experimental tar

**Figure 1:**
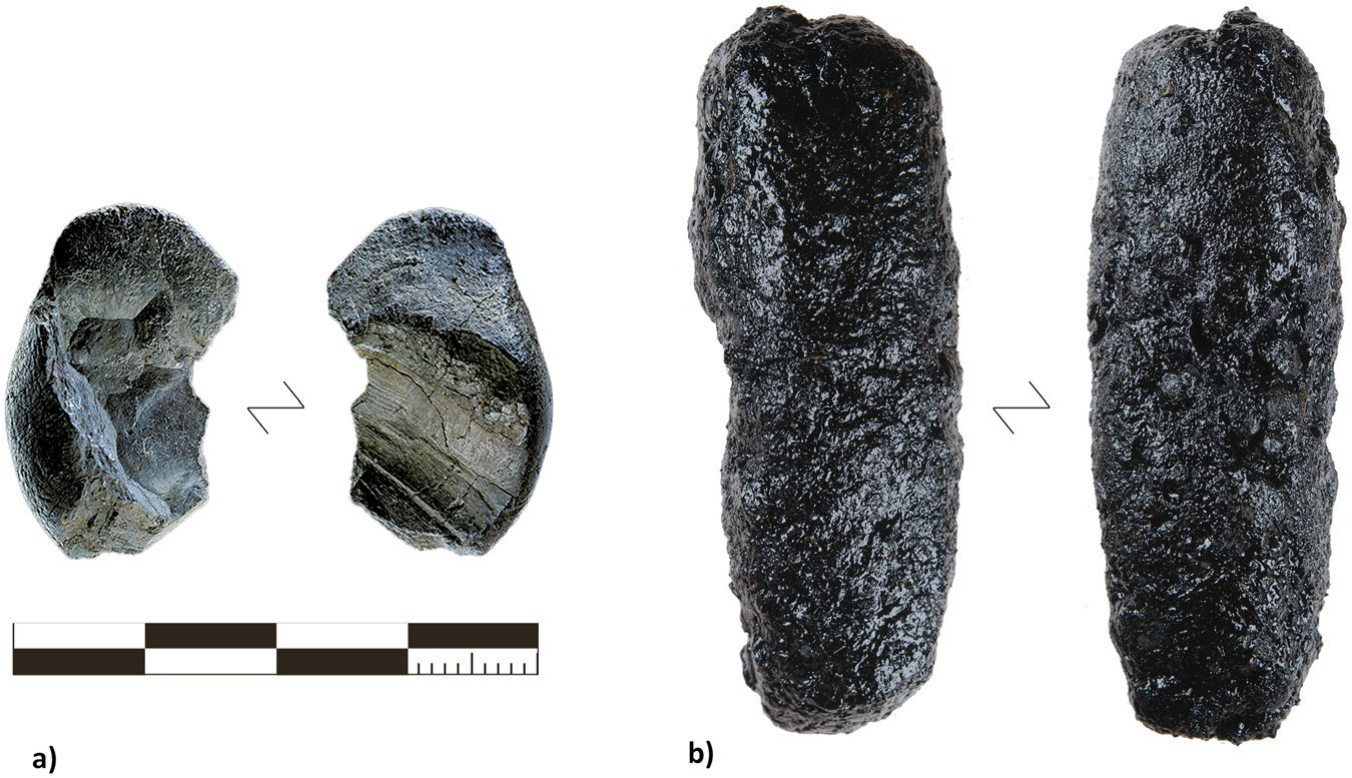
(**A**) The larger of the two tar lumps found at Königsaue (photo credit: Landesamt für Denkmalpflege und Archäologie Sachsen-Anhalt, Juraj Lipták) compared with (**B**) the maximum yield of tar produced with the raised structure method (RS 7).

The tar we produced was a dark brown/black material that varied in consistency somewhat depending on the method. We use the term ‘tar’ here rather than ‘pitch’ because our experimental products varied in consistency depending on the method and ambient temperature. Tar more accurately describes the complete material initially produced during destructive distillation, while pitch is generally more solid, and may require further refinement^29^. The ash mound produced tar tended to be the hardest, as many of the liquids﻿ and volatiles can easily escape during production due to the porosity of the ash. The pit roll and raised structure methods produced softer materials. They also contained only slight charcoal and soil contamination. All of the experimental tars would be suitable for hafting at the ambient temperature they were produced at (~5 °C), but the pit roll and raised structure tars became somewhat softer at room temperature (Fig. 1).

The tar yield described below uses data from our most successful experimental attempts. This reduces any potential bias that may exist due to our own skills and learning curve. There is very little modern expertise regarding producing birch bark tar aceramically. Our results indicate a starting point, and should not be considered the maximum possible output rate, or be used to directly interpret how long it would take Neandertals to make tar. All the data from our experiments are provided in the Supplementary Information to help reproducibility and explain in detail what the values represent.

### Ash mound

Up to approximately 1.0 g of tar per 100 g of bark was obtained using the ash mound technique. Ambers and ash were placed over a bark roll, tied with fresh wood fibre to keep it tight^19^. No vessel, pit or structure is required using this technique. Tar was collected between the bark layers and could be scraped off (Supplementary Fig. S1). However, because the roll was in direct contact with embers from a glowing fire, care needed to be taken to balance the ratio between embers and ash. Ash keeps the oxygen out, but too much will lower the temperature. Likewise, too many embers can raise the temperature and oxygen content and tar will burn before being collected.

### Pit roll

Techniques similar to the one described by Pawlik^23^, in which a roll of bark is ignited and placed burning side down into a small pit with a pebble at the bottom to collect the tar, were found to be unsuccessful. The temperature was never high enough or sustained for a long enough period of time to produce tar (Supplementary Fig. S2). The pebble used to collect the tar was blackened due to the burning roll being placed on top, but no tar was found. Rather than placing the burning end in a pit, we were successful when hot embers were placed on top of the bark to provide continuous heat. Pyrolysis oils and tar dripped out of the bottom of the bark roll in small quantities, and in one case (PR11) a considerable amount of tar (1.8 g) was collected in the birch bark vessel placed below the roll (Supplementary Fig. S3). In some experiments tar was also collected from between each layer of bark in a similar manner to the ash mound method. Using the pit roll technique with capping embers and bark container, the maximum tar output was 2.4 g per 100 g of bark.

### Raised structure

**Figure 2:**
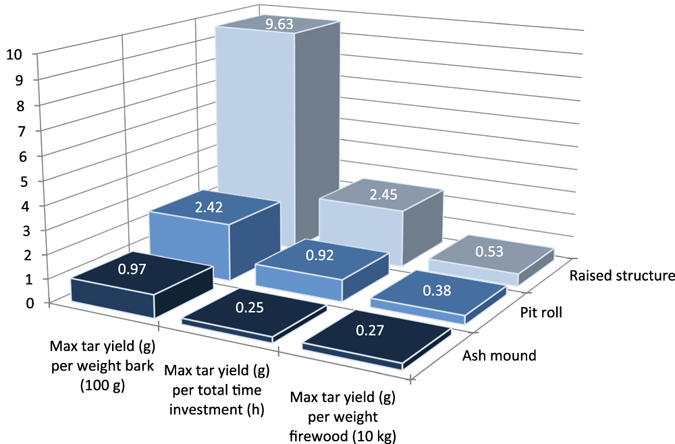
Maximum tar production efficiency for each method tested. If ash and embers from a fire used for other tasks were utilized then the tar yield/time investment and tar yield/firewood for the ash mound and pit roll method would also increase.

Here we adapted a method described by Groom and Schenck^17^; a birch bark container was placed in a pit, an organic mesh covered the pit, and on the mesh we placed a large loose roll of bark. The bark was then covered with earth and a fire was lit over the mound (Supplementary Fig. S10). This method resulted in the most variable output of tar, but when successful it gave the highest yields by a large margin (Fig. 2). Despite requiring the longest set-up and run-time, as well as using the most firewood, it was the most successful and efficient method. We achieved a maximum tar yield using this technique of 9.6 g per 100 g of bark, or a total of 15.7 g from one attempt.

### Comparison with archaeological tar

The three largest prehistoric birch bark tar finds are those from the Middle Palaeolithic sites of Campitello Quarry in Italy^9^, Königsaue in Germany^30^ and the Mesolithic site of Star Carr in England^31^. Using a value of 1.14 g/ml for the density of wood tar^29^, the largest volume of birch bark tar found at Campitello Quarry measuring approximately 40 × 32 × 18 mm should weigh a maximum of 14.6 g, not excluding the volume occupied by a ~5 mm thick flint flake. The smaller residue from Campitello Quarry is less than 20 × 20 mm and only a few mm thick, but this is likely incomplete^9^. Due to degradation, the values of 1.38 g and 0.87 g given for the tar found at Königsaue^30^ are unlikely to represent the original mass of the lumps. These must have been closer to 5.7 g and 1.7 g given the known density of wood tar^29^ and the dimensions of the lumps^30^. The tar finds from Star Carr, described as ‘resin cakes’, are between 25 mm and 45 mm in diameter and a few mm thick^31^, so were likely originally between 1.5–6.5 ml, or 1.7–7.5 g.

These volumes are well within the production range of all our methods. For some of the most successful runs, we produced approximately 1.0 g of tar from the ash mound, and 1.8 g of tar from the pit roll. These would therefore need to be repeated only once or twice to produce the smaller lump of tar from Königsaue, and between six and 11 times to produce the tar found at Campitello quarry. If the ash and embers for the ash mound and pit roll methods were obtained from a central hearth used for cooking and/or other purposes, then the efficiency is improved and having to repeat this process would not be much of a drain on fuel resources. Alternatively, our raised structure method produced 15.7 g of tar in one successful attempt, enough to make a ‘cake’ or lump nearly 45 mm in diameter and 10 mm thick, as large as those found at Star Carr^31^, Campitello Quarry^9^, or larger than both lumps found at Königsaue combined (Fig. 1). It is also worth considering that our own hands-on practice was limited and improved across time for the pit roll and raised structure techniques (Supplementary Table S1). We in turn expect that with more practice the tar yield will improve further.

If tar was produced on an opportunistic basis, when there was a fire present, when a single tool required repair, or when limited time was available, the plausibility of using simpler low-yield methods increases. It is also possible that the archaeological examples of tar have survived, or more likely have been recognized during excavation, because they are exceptionally large. A tightly fitted haft, or a joint that also contains a binding will require less tar than that found at Campitello. This combined with the ideas that adhesives can be reused, and that it is unlikely a Neandertal would need to haft an entire toolkit at once, further demonstrate the feasibility of the methods used here.

Depending on the tree species, tar yields using laboratory techniques are in the range of 3.1% (*Quercus cerris*) to 14.3% (*Betula alba*)^32^, so our yield of 9.6% using the raised structure is comparable even to dry distillation in a lab setting using glass containers. Moreover, our tar is naturally more condensed than lab produced tar which retains all volatiles; if lab produced tar were to be reduced to a semi-solid suitable for hafting, the yield would decrease further and be even closer to what we attained. All of the aceramic methods tested here are therefore viable in terms of yield and what is known from the archaeological record.

### Temperature control

**Figure 3:**
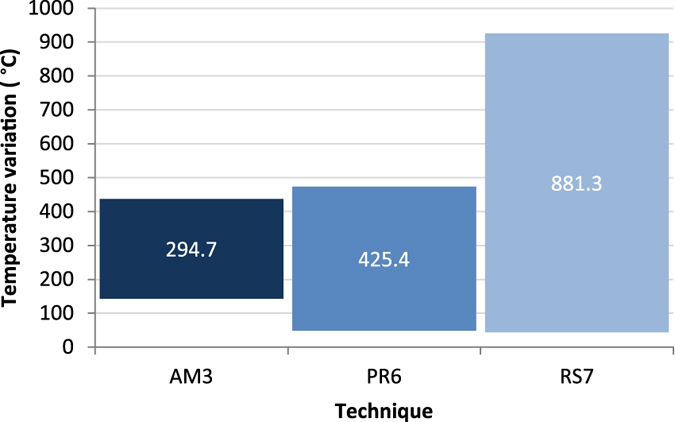
Display of temperature variation within each method. The temperature inside the bark roll (AM3) and vessel (PR6, RS7) was recorded when the temperature in the heat source (fire or embers) was at its its maximum. This provides an estimate of the range of temperatures that can exist at a single point in time for each method

During our successful (tar-yielding) experiments there was at least one point for each method (either in the fire, ashes, or bark) that exceeded 400 °C, and another point (in the bottom of the roll or pit) that was less than ~200 °C. Between these two points conditions are suitable for tar production; for birch bark this can be as low as 250–300 °C^33^ and over 500 °C^34–36^. For the ash mound technique, maximum and minimum temperatures between the inside and outside of the bark roll varied relatively little compared with the other methods (Supplementary Fig. S5). In the raised structures, fire temperatures fluctuated dramatically and reached as high as 900 °C, but the structure kept the birch bark closer to 450 °C or less and the collection vessel below 150 °C (Supplementary Figs S6 and S7). Temperatures for the pit roll technique are intermediate with the hottest temperature in the bark and the coolest temperature in the pit itself. The vessel in the bottom of the pit never reached more than 100 °C (Supplementary Fig. S8). The ability to strictly control temperatures to a narrow range between 340 °C and 370 °C for tar production^6, 10^ is thus not as necessary as previously thought (Fig. 3).

The degree of temperature monitoring also appears to vary directly according to the complexity of the structure. Actualistic fire experiments have shown that surface fire temperatures can fluctuate dramatically, while sub-surface temperatures below a fire are more constant^37^. Due to the direct contact that the birch bark roll has with hot embers and oxygen in the ash mound, this method is more similar to a surface fire and the temperature needs to be managed more closely. Here small amounts of ash were added to the mound if it appeared to be smoking too much, and embers were added if it seemed too cold, although this was subjective and relied only on the operator’s experience. It was clear during our experiments that the operator with the highest hands-on experience with the ash-mound technique (author DP^19^) produced the most consistent amount of tar (Supplementary Table S1). On the other hand, with the raised structure method, the structure itself manages the temperature by isolating the bark from the fire, thus removing this level of know-how from the equation; all that is needed is to maintain flaming combustion around the structure. This would have required the same level of attention as tending a hearth for purposes such as warmth, light, or cooking. However, because the flames needed to be burning for several hours, this process would have required more effort and attention to collect wood and maintain the fire than the ash mound or pit roll method. As with previous experiments^16^, it seems that once learned, this method is simple to operate. In terms of required temperature control, the pit roll method falls between the ash mound and the raised structure technique. Just as the sub-surface temperature in an open hearth is lower and more controlled than the surface temperatures^37^, the temperature in the pit is lower and more stable than the ash and embers above the pit. The tar will never burn away completely because the depth of the pit limits the oxygen to such an extent that the temperature begins to decline automatically before getting too hot (Supplementary Fig. S8). Using this method, bark and embers could be put in place, and the process could be left alone without requiring any further intervention or attention. The only significant limitation is that if the embers are too small to begin with they may burn out before much tar is produced.

### Complexity

**Figure 4:**
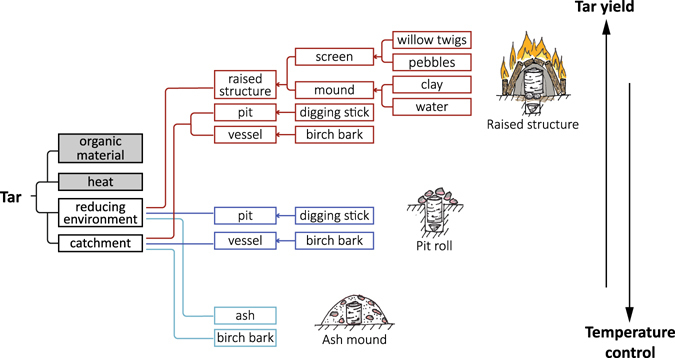
Depiction of the increase in complexity of each method and the associated increase in tar yield and decrease in required temperature control.

The setup time and the run time of each method increased in the same order as the number of steps and the material diversity. Excluding tools and processes required for fire production, the ash mound is made of the fewest individual components (embers, ash, and birch bark). The pit roll method requires more components (digging stick, vessel, pit, embers, birch bark), and the raised structure method requires yet more components (digging stick, vessel, pit, willow twigs, pebbles, earth, water, fire, and birch bark). If we use the maximum yield obtained for each method (Fig. 2) the results indicate that as the complexity increases so does the amount of tar obtained (Fig. 4).

The amount of temperature control required is also directly associated with the structural complexity of each method. As more complex techniques are employed, the amount of oxygen is reduced and the bark is isolated. The control of heat is thus ‘automated’ by the structure, reducing the practical expertise required to control the temperature while increasing tar yield (Fig. 4). This pattern is repeated in historical and modern tar and charcoal production techniques as well. Internally heated tar pits or mounds (in this case similar to our ash mound) have relatively few separate parts, but require constant care by numerous people to manage the internal environment during the entire firing process^38^. The introduction of kilns, although more complex structurally, required less manual or personal management and improved yields^26, 38^. The implementation of various modern feed-stock gas furnaces takes this one step further by completely automating the process^39^.

## Discussion

It is known that Neandertals were fire users, even if the necessity of fire use and their ability to produce fire on demand is disputed^40–42^. Here, we show that tar can be produced using aceramic technology compatible with a Neandertal context. Enough tar can be produced using low-tech solutions, such as the ash mound with the only prerequisites being the presence of fire and birch bark.

### Origin of tar technology

Birch bark is an excellent fire starter^43, 44^, tends to roll naturally when peeled off a tree, will curl further on exposure to heat, and is known in the ethnographic record to have been used (rolled) as torches and fire-lights^27, 45, 46^, as well as to have served many other practical purposes^46, 47^. Birch bark is known to contain proportionally higher extractives than many other plant resources^32, 48, 49^, and was also one of the most common trees in Palaeolithic Europe^50, 51^. A tightly rolled piece of birch bark simply left in a fire and removed when partially burned, once opened, will sometimes contain small traces of tar inside the roll along the burned edge. Not enough to haft a tool, but enough to recognize a sticky substance. From this point the ash mound is a small step forward. Piling the remnants of a hot fire over the bark is also analogous to some traditional cooking methods using ash^52, 53^ (see also ref. 54).

Hafting technology is known from 300-200 ka and may be as old as 500 ka^55^. Neandertals are known to have used wood as a resource^56–58^, remains of birch bark charcoal have been identified^59, 60^, and fire use did occur during the middle Pleistocene^59–61^. To produce tar using the ash mound technique would only necessitate the combination of materials and properties already known by Neandertals. It is therefore not surprising that Neandertals discovered how to produce birch bark tar and used it for hafting.

The largest imaginative leap required to use tar for hafting would have been the comprehension of using a sticky substance to hold two objects together. However, early forms of hafting, possibly without adhesives, may predate the discovery of birch bark tar^62, 63^. Water resistant materials, such as fats, resins, and tars can also be used to protect bindings from moisture^64^. It is possible that the early function of tar may have been to assist and waterproof the binding on a haft (e.g. sinew, hide, or vegetal fibers), and as the production and quantities of tar improved, it gained the more primary function as a fixative agent or adhesive.

### Development of tar technology

We can hypothesize that after having discovered tar while using birch bark close to a fire-place, a major improvement will be placing a bark roll in a depression or pit to limit the oxygen and prevent too much of the tar or bark from burning away. It could have then been observed that pyrolysis products would flow out of the bottom of the bark roll, so a catchment method would further improve the yield. Yet, using this method, some tar and bark are still lost to combustion. The third major improvement would be to isolate the bark from direct contact with extremely high temperatures and oxygen by building a clay or earthen structure. Placing the bark inside an enclosed structure with the heat source outside reduces the likelihood of tar or pyrolysis products from burning away. Creating a screen to support the bark and raising the bark and structure above ground aids in heat transfer. The pit allows for vessels of non-heat-resistant materials to be used, and prevents the tar from being exposed to excessive heat for a prolonged period. Our own methods are combinations and improvements on previously tried techniques, both historical and experimental, and it is likely that numerous other combinations or variations could exist to fill in the gaps.

The discovery of birch bark tar can be explained through a number of discrete technological steps, rather than requiring any major *eureka* moment or leap of innovation. This also increases the possibility for the independent discovery or re-discovery of this technology throughout the Middle Palaeolithic. To acquire the necessary expertise to produce useable quantities of tar, however, Neandertals must have been able to recognize properties, such as adhesive tack and viscosity. In this way they could develop the technology from small traces of tar on partially burned bark to techniques capable of producing the volumes required to haft a large stone flake.

### Possible archaeological traces

Lack of adhesive evidence during the Middle Palaeolithic may be a product of taphonomic or research biases, so understanding what to look for will be beneficial to future studies. Unfortunately traces of early tar production strategies are unlikely to be easily discernible in the archaeological record. The ash mound method leaves virtually no trace, and the only remains from the pit roll method were a small depression less than 10 cm deep by 10 cm in diameter. Although the centre of the bark roll reached high enough temperatures to leave a lasting trace in the soil, the bottom of the pit did not^65, 66^.

One of the most enduring traces could have been the pebbles, yet our experiments showed that their use for collecting or ‘condensing’ tar is not necessary. We found that in many cases a birch bark vessel was in fact the best option. It was never so hot that a fire-resistant retort was required, and the funnel shape available from a folded circular piece of bark allowed for the collection of greater quantities of tar. Tar removed from these birch bark vessels also contained traces of un-charred bark. The presence of un-charred bark to describe an incomplete production process^23^ must therefore be used with caution as it may in fact come from successful attempts.

If the earliest tar-makers, whether it was at Campitello Quarry, Italy^9^ and Königsaue, Germany^10^, or at some still undiscovered archaeological site, used simple techniques then it will be difficult to find direct traces of the first tar production strategies. However, the tar lumps themselves may be able to give further insights into the evolution of the technique used. Chemical and microscopic analysis of experimental material alongside archaeological remains may help illuminate which methods were likely used in the past by understanding the formation and thermal degradation of biomarkers (cf. refs 10, 67) and by identifying additives.

## Conclusion

While there are many potential methods of producing tar^16, 19, 20, 25, 26^, we have demonstrated that there are at least three successful aceramic solutions, ranging from low to high-tech. A simple bark roll in hot ashes can produce enough tar to haft a small tool, and repeating this process several times (simultaneously) can produce the quantities known from the archaeological record. Our experiments allowed us to develop a tentative framework on how the dry distillation of birch bark may have evolved, beginning with the recognition of small traces of birch bark tar in partially burned bark rolls. Small changes and additions to the production process would have allowed easier regulation of fire temperatures, and improved tar yield efficiency. Such a framework is consistent with the technology and resources available to Neandertals during the Middle Palaeolithic. Given the ephemeral nature of the expected traces, however, it will be difficult to find direct evidence for the evolution of tar production techniques in the sediments of Palaeolithic archaeological sites. Further investigation of the composition and nature of the tar lumps themselves may help to refine the history of the development of tar technology.

Considering that birch bark was available in Europe during the Pleistocene, and that Neandertals are known to have used wood resources and fire, it is now clear that Neandertals could have invented the transformative technology simply by recombining knowledge they already had. Such an invention must have been driven by curiosity and interest in properties like the tack and viscosity of the newly discovered material. Moreover, in order for tar production to become a perennial innovation, Neandertals must have been able to maintain the process of dry distillation as a useful technique for producing adhesives.

## Methods

### Materials

Birch bark from *Betula pendula* trees was collected in southern England and the Netherlands during August 2016 and prepared into rolls on-site before each experiment in December 2016. Bark from both branches and trunks of trees ranging from approximately 5 cm to 15 cm in diameter was used. Firewood consisted of store-bought kiln dried assorted European hardwoods (*Quercus sp., Fagus sp.*, and *Fraxinus sp.*) with a moisture content of approximately 10–15%. Pollen records show oak (*Quercus* sp.) was present in Europe at times associated with the use of birch bark tar^50, 68^ and all three of the firewoods used have calorific values comparable to birch. The greatest variation in thermal output of firewood comes from moisture content^69^, which we controlled by using kiln dried woods. Experiments were conducted under a shelter at the Leiden University experimental house at the Horsterwold in Flevoland, the Netherlands. A weather station (Alecto WS4050) was placed several meters away to record the local ambient temperature, humidity, and wind speed and direction during each experiment. Temperatures during tar production were recorded at several points for each method using thermocouples connected to an Extech SDL200 4 channel temperature meter (Supplementary Figs S9–S11). The thermocouples were not consulted to guide the experiments; the collected data was only used for analysis after the experiments were complete. A breakdown of the three tar production methods tested is described below.

### Aceramic distillation experiments

Three tar production methods were used, and each was tested between 5 and 11 times (Supplementary Table S1). For each experiment, set-up time, run-time, fuel use, temperature curves, technounits^27^, operational steps and tar yield has been recorded. Details and photographs of the remains from each method are available in the Supplementary Information.

### Ash mound

A tightly made roll of birch bark (approximately 10 cm long by 7 cm diameter) was covered in embers and ash from a long-burning fire^19^ (Supplementary Fig. S1). The heat from the embers works with the ash and the tightly rolled bark to limit oxygen, inhibiting combustion and encouraging the formation of tar. No vessel was used and the tar was scraped off each consecutive layer of bark as the roll was unwrapped^19^.

### Pit roll

The pit roll method involved digging a small cylindrical pit, in this case approximately 8 cm deep by 6 cm in diameter to help exclude oxygen. A bark roll (approximately 9 cm long by 5 cm diameter) was placed inside the pit. We tested three principle variations of this method. PR1 and PR2 were based on the description given by Pawlik^23, 24^. A pebble was placed in the bottom of a pit, and a roll of birch bark was ignited. The burning end of the bark was then placed into the hole. PR3 and PR4 are similar, but with the burning end up to try and encourage longer combustion. PR5-PR9 had hot embers placed on top of the bark in order to provide additional heat. PR5 contained a pebble in the bottom of the pit, PR6 contained a strip of bark in the bottom of the pit, and PR7-PR9 used a small birch bark cup tucked in the bottom of the roll to collect tar and pyrolysis oils that dripped out of the bottom of the bark (Supplementary Fig. S3).

### Raised structure

This method was essentially a reproduction of the ‘two pot’ method^70^ without the use of metal or ceramic containers (Supplementary Fig. S4), although we did use a metal collection container in one attempt. A small pit was dug in the ground (approximately 7 cm deep and 9 cm wide) and a vessel made of birch bark was placed at the bottom of the pit. A screen of green willow wood (*Salix* sp.) sticks was placed across the top of the pit, pebbles and then a roll of birch bark (approximately 15 cm long by 15 cm diameter) was placed on top of the screen. Wet earth was placed over the bark to seal the bark inside a dome-like structure.

### Data Availability

All data generated or analysed during this study are included here and in the Supplementary Information files.

## Electronic supplementary material

Supplementary Information
