# Supplementary material for: Experimental methods for the Palaeolithic dry distillation of birch bark: implications for the origin and development of Neandertal adhesive technology
Source: Sci Rep. 2017 Aug 31;7:8033. doi: 10.1038/s41598-017-08106-7 (PMC5579016; doi:10.1038/s41598-017-08106-7)
Supplement: Supplementary file 1 — Supplementary Information [file 41598_2017_8106_MOESM1_ESM.pdf]

Supplementary Information

Experimental methods for the Palaeolithic dry distillation of birch bark: implications for the origin and development of Neandertal adhesive technology

P.R.B. Kozowyk [1]*, M.A. Soressi,[1] D. Pomstra, G.H.J. Langejans[1,2]

1. Faculty of Archaeology, Leiden University, the Netherlands

2. Department of Anthropology and Development Studies, University of Johannesburg, South Africa

* Corresponding author: p.r.b.kozowyk@arch.leidenuniv.nl

Supplementary Table S1. Recorded data for each attempt.

Exp.	Fire prep time (min)	Set-up time (min)	Run time (min)	Firewood (kg)	Bark before (g)	Bark after (g)	Tar yield (g)	Tar yield efficiency (%)	Ambient temp (°C)	RH (%)	Wind speed (Km/h), direction	Notes
AM 1	120	17	32	10.30	88.00	33.10	0.31	0.35	5.2	91	1.1, SE	
AM 2	180	10	19	7.10	98.00	62.54	0.59	0.60	5.6	92	2.5, S	Firewood added to ash and embers from previous fire
AM 3	210	10	30	3.90	107.00	49.72	1.04	0.97	5.5	92	1.1, SE	Firewood added to ash and embers from previous fire
AM 4	310	10	28	2.90	120.00	54.13	0.18	0.15	4.7	96	0.0, -	Firewood added to ash and embers from previous fire
AM 5 Demo	300	10	36	N/A	53.00	20.45	0.36	0.68	1.2	97	0.0, -	Firewood not recorded
**Mean:**				**6.05**	**93.20**	**43.99**	**0.50**	**0.53**	**4.4**	**93**		
PR 1	10	22	5	1.00	32.00	30.00	N/A	N/A	5.5	6	3.0, SW	Unsuccessful
PR 2	10	10	5	1.00	30.00	29.00	N/A	N/A	5.5	6	3.0, SW	Unsuccessful
PR 3	10	10	8	1.00	50.00	51.24	N/A	N/A	5.4	6	3.6, SW	Unsuccessful
PR 4	10	5	4	1.00	47.00	43.65	N/A	N/A	5.4	6	3.6, SW	Unsuccessful
PR 5	60	14	30	4.00	52.00	32.67	0.13	0.25	5.0	67	0.0, -	
PR 6	60	15	33	4.00	45.00	26.71	1.53	3.40	2.5	67	0.0, -	High soil contamination in sample
PR 7	60	15	30	4.00	48.00	23.34	0.80	1.67	6.2	75	1.1, SE	High soil contamination in sample
PR 8	60	15	40	4.00	63.00	44.32	0.11	0.17	6.5	73	2.5, S	
PR 9	60	15	58	4.00	50.00	33.38	0.12	0.24	5.4	79	0.0, -	
PR 10	60	15	41	4.00	62.00	28.03	0.15	0.24	10.6	88	1.1, SE	
PR 11	60	15	40	4.00	73.00	33.41	1.77	2.42	11.2	82	1.1, SE	
**Mean:**				**4.00**	**56.14**	**31.69**	**0.66**	**1.17**	**6.7**	**90**		
RS 1	10	45	150	7.50	85.00	78.46	N/A	N/A	3.8	99	0.0, -	Unsuccessful - some tar soaked into wood vessel (not enough bark)
RS 2	10	15	165	8.80	88.00	47.34	N/A	N/A	5.4	96	0.0, -	Unsuccessful - some tar soaked into wood vessel (not enough bark)
RS 3	10	20	180	10.70	52.00		N/A	N/A	5.5	92	0.0, -	Unsuccessful - some tar soaked into wood vessel (not enough bark)
RS 4	7	21	230	11.20	52.00	34.31	5.52	10.62	3.0	96	0.0, -	Metal vessel
RS 5	8	22	235	11.20	57.00	39.90	N/A	N/A	3.0	96	2.5, NW	Unsuccessful - some tar soaked into wood vessel (not enough bark)
RS 6	10	25	210	13.00	55.00	6.89	0.17	0.31	5.4	71	2.2, S	Likely not enough bark
RS 7	20	25	340	29.50	163.00	35.00	15.70	9.63	1.2	97	0.0, -	
RS 8	10	57	376	23.90	194.00	38.00	1.86	0.96	8.3	94	0.0, -	Shell vessel - high soil contamination
**Mean:**				**15.22**	**93.25**	**39.99**	**5.81**	**6.23**	**4.4**	**92**		

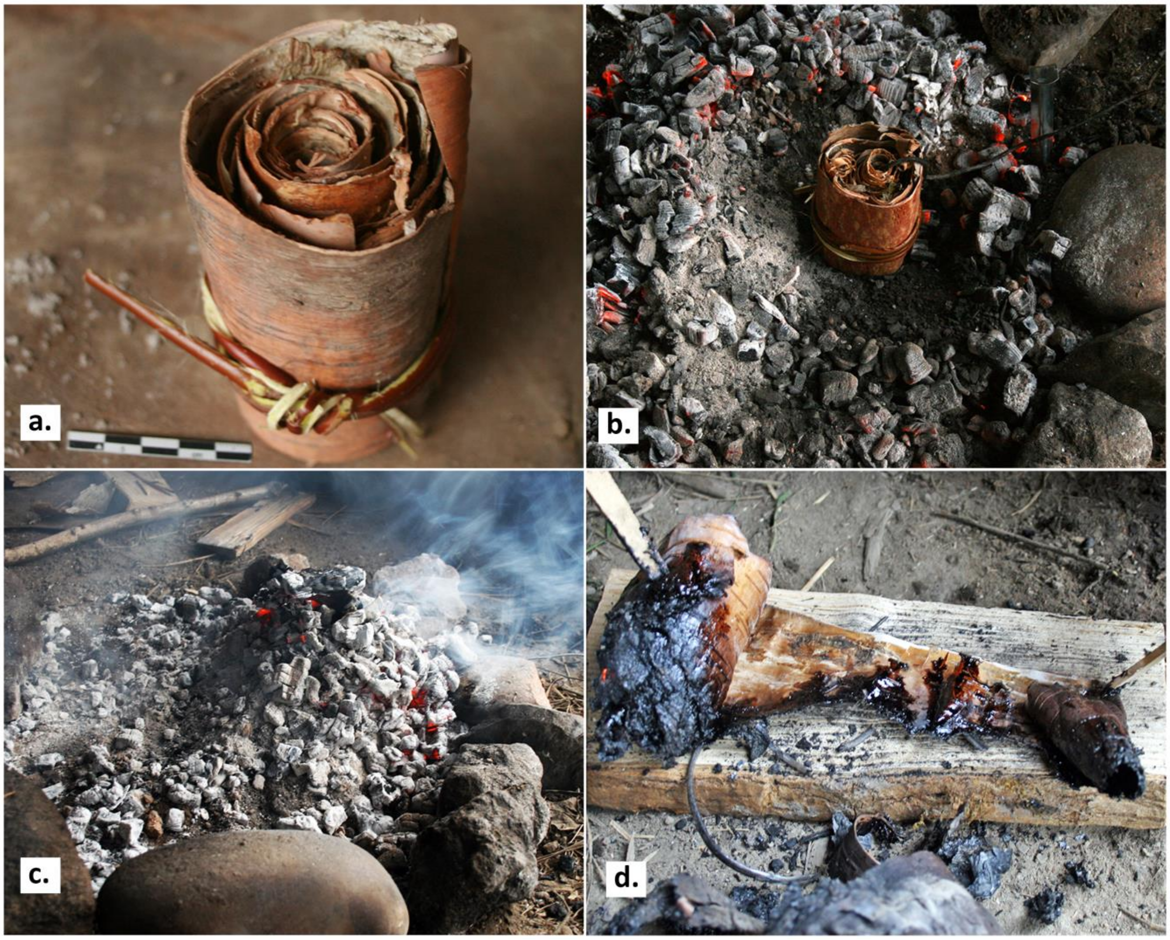

Supplementary Figure S1. Photographs of ash mound method. A) Birch bark roll; b) Birch bark roll placed in embers and ash; c) Birch bark roll covered in pile of embers and ash; d) Unwrapping birch bark roll to expose tar which is then scraped off using a stick or flint flake.

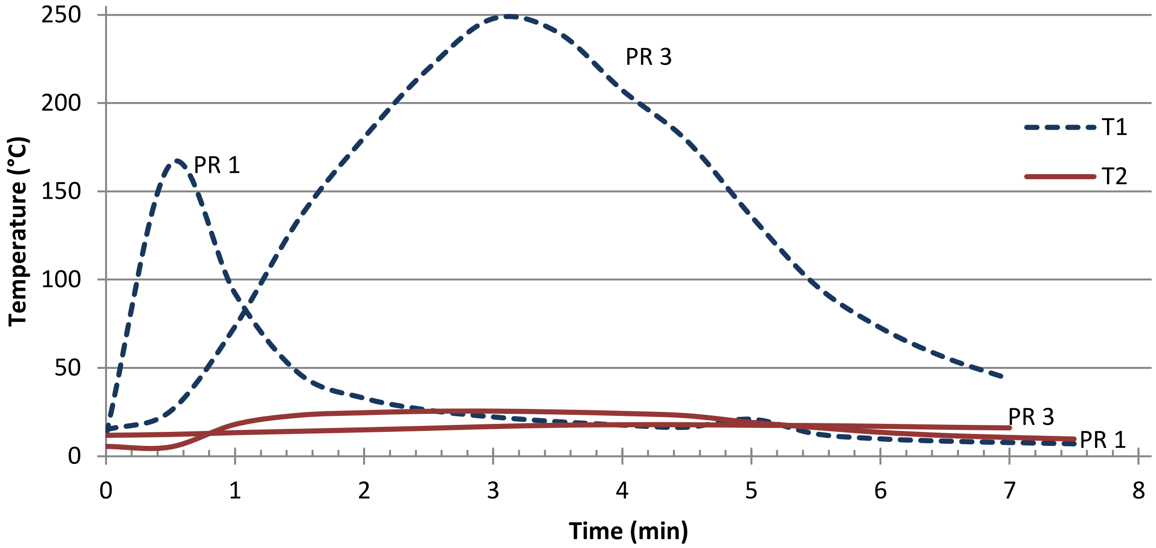

Supplementary Figure S2. Temperature curves of pit roll methods PR1 and PR3 using a pebble and no additional heat source. Temperature was not sustained high enough or long enough to produce tar. T1 = thermocouple in the middle of the bark roll; T2 = thermocouple in the pit below the bark roll.

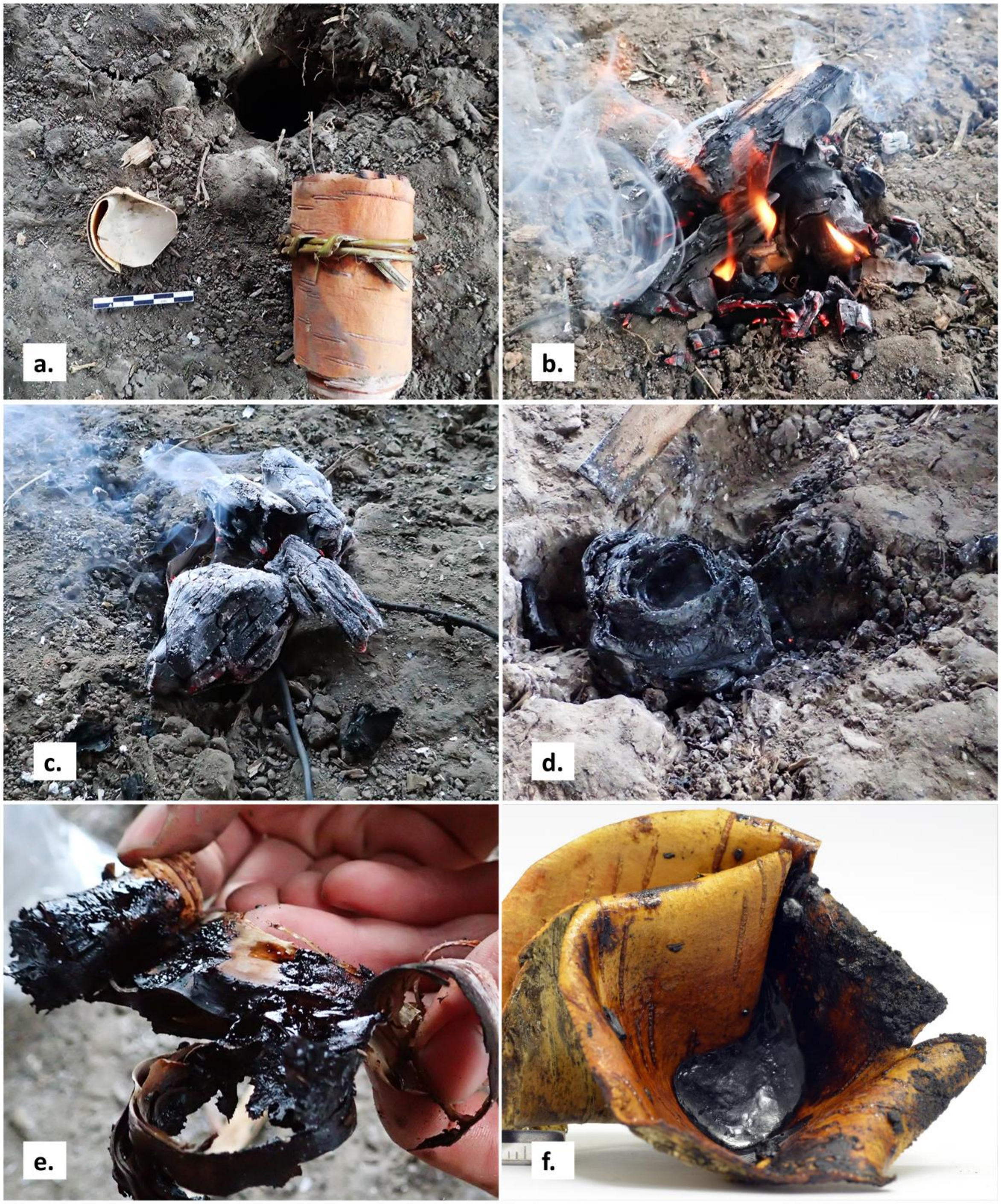

Supplementary Figure S3. Photographs of pit roll method. a) Birch bark roll, pit, and birch bark vessel; b) birch bark placed in pit with hot embers covering everything (flames sometimes occur near the start); c) embers smouldering over the birch bark in pit; d) embers are removed to reveal charred bark (on the top half); e) bark is unrolled to expose more tar, some of which has dripped out of the bottom of the roll and into the vessel in the bottom of the pit; f) birch bark vessel from the bottom of the pit with birch bark tar.

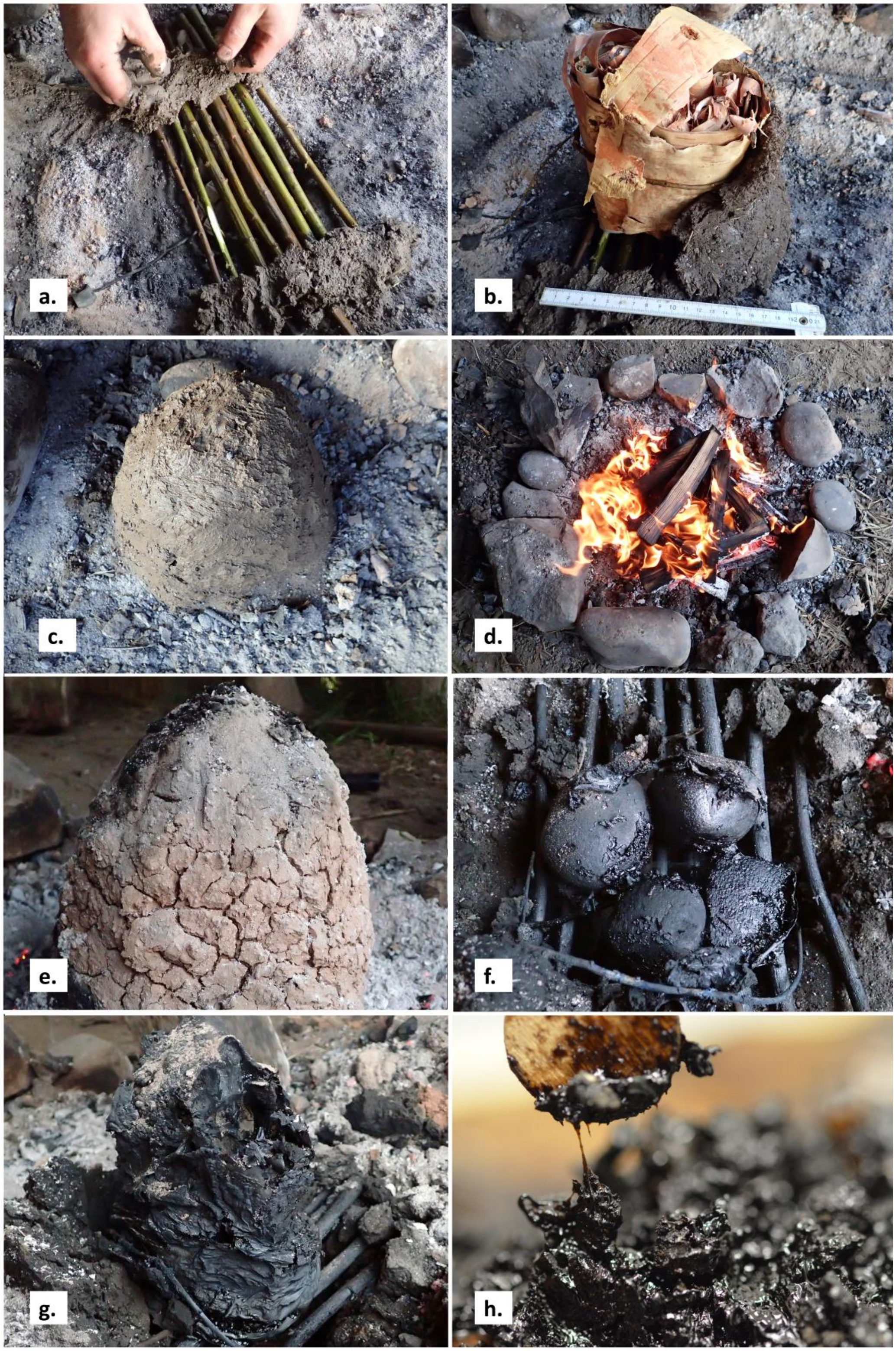

Supplementary Figure S4. Photographs of raised structure method. a) Twigs laid over pit containing birch bark vessel; b) and c) large roll of bark covered in wet clayish soil; d) fire lit all around raised structure; e) structure after firing; f) and g) structure removed to expose pebbles, charred twigs, and charred bark; h) resulting tar scraped from vessel at room temperature.

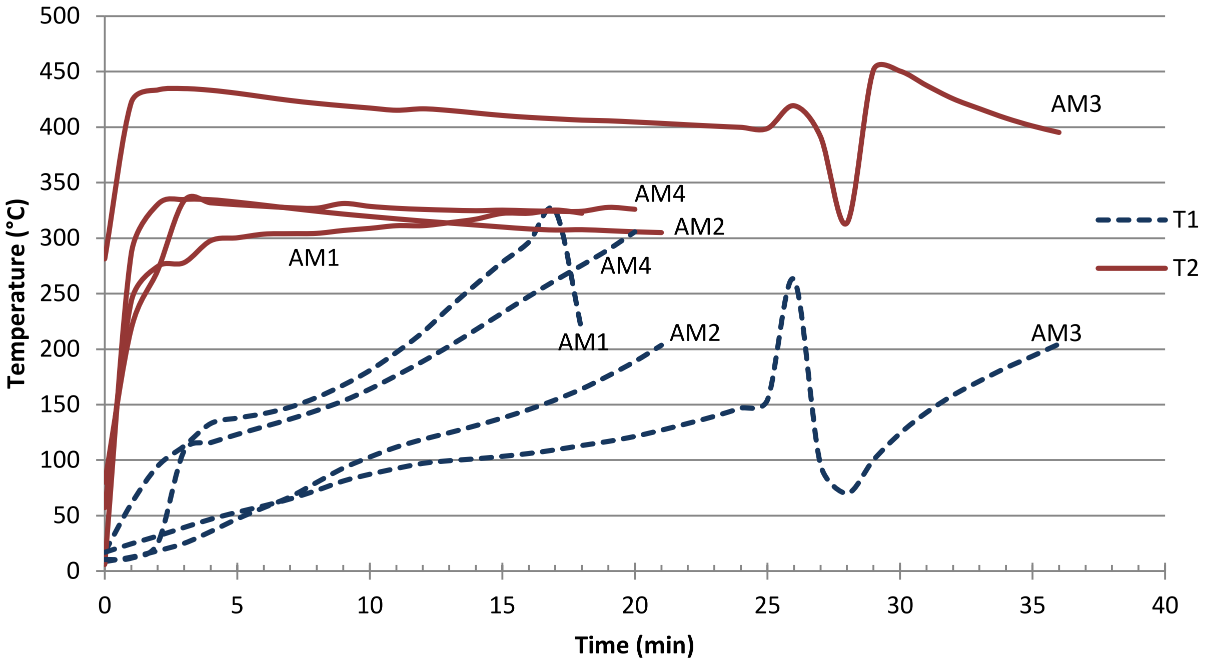

Supplementary Figure S5. Temperature curves for ash mound methods AM1-AM4. The temperature fluctuation of AM3 between 25 and 30 minutes occurred when we removed the bark roll from the ash/embers and placed it back in the fire. T1 = thermocouple in the middle of the bark roll; T2 = thermocouple in the ash/embers outside the bark roll.

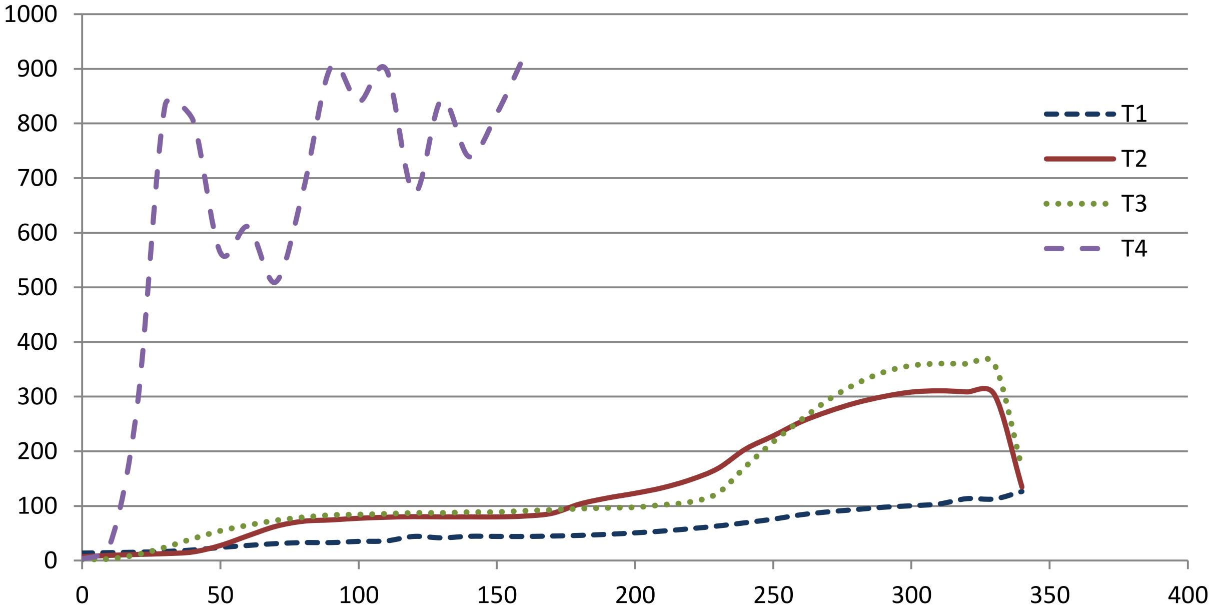

Supplementary Figure S6. Temperature curve for Raised structure RS7. Temperatures inside the earth structure remain below 100 °C until the water has evaporated and then begin to climb between ~175-225 min. Fire temperatures fluctuate dramatically from very early on. In this experiment the fire thermocouple was removed at 170 min due to time constraints on the equipment, but the fire was continually fed until ~325 min. T1 = thermocouple in the vessel in the pit; T2 = thermocouple in the middle of the bark roll; T3 = thermocouple outside the bark roll but inside the earth structure; T4 = thermocouple in the fire.

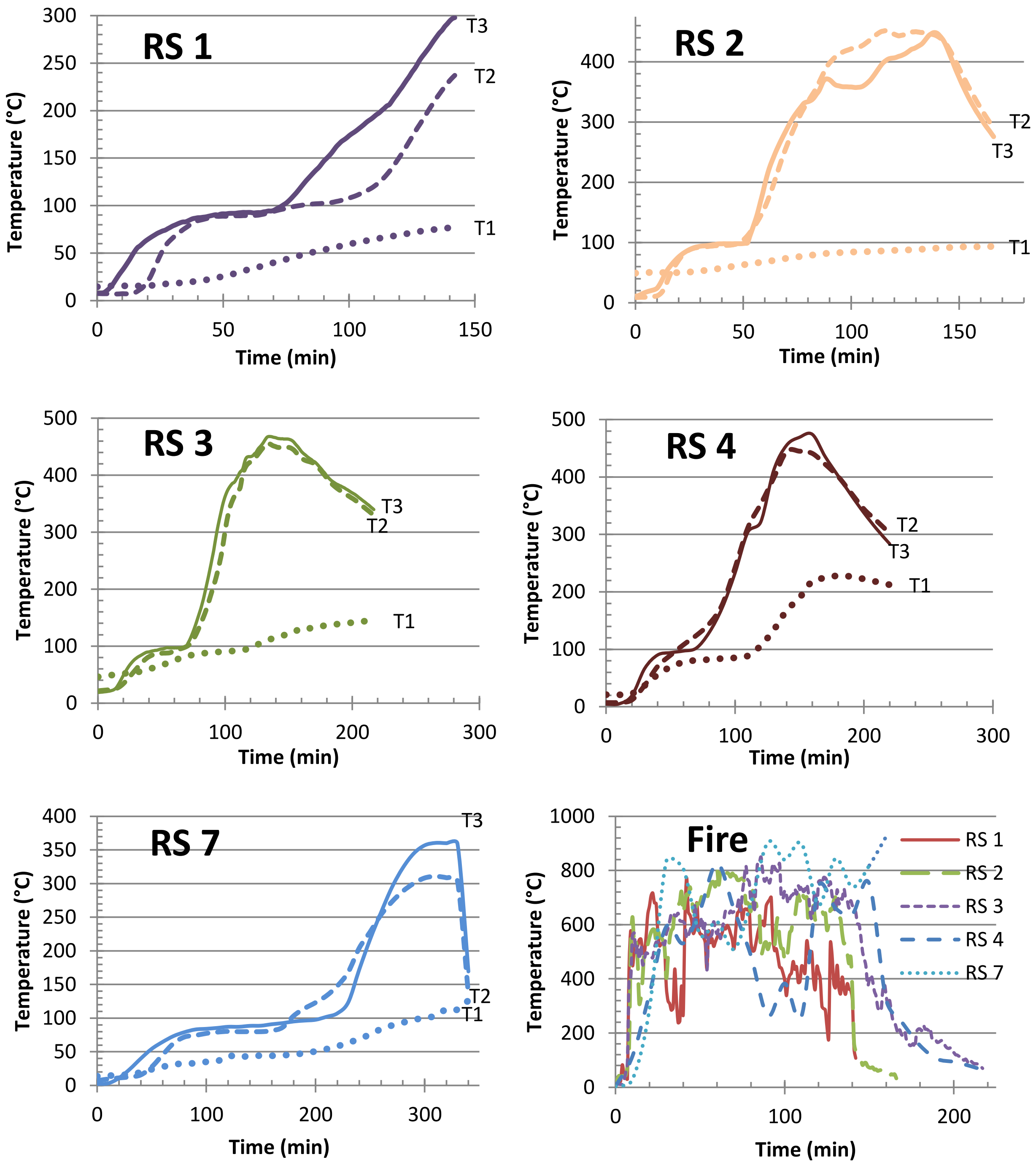

Supplementary Figure S7. Temperature curves of raised structure experiments. RS 1-4 T1= vessel, T2=inside middle of roll, T3=outside middle of roll. RS 7 T1=vessel, T2=inside bottom of roll, T3=inside top of roll. The first 50-80 minutes is spent evaporating the moisture from the earth mound, seen by the plateau in temperature for the first part of the curve. In RS7 the larger structure took nearly 200 minutes to dry before heat inside began to climb over 100 °C. Highly fluctuating fire temperatures and steady internal temperatures indicate automatic thermo-regulation by the clay structure and pit.

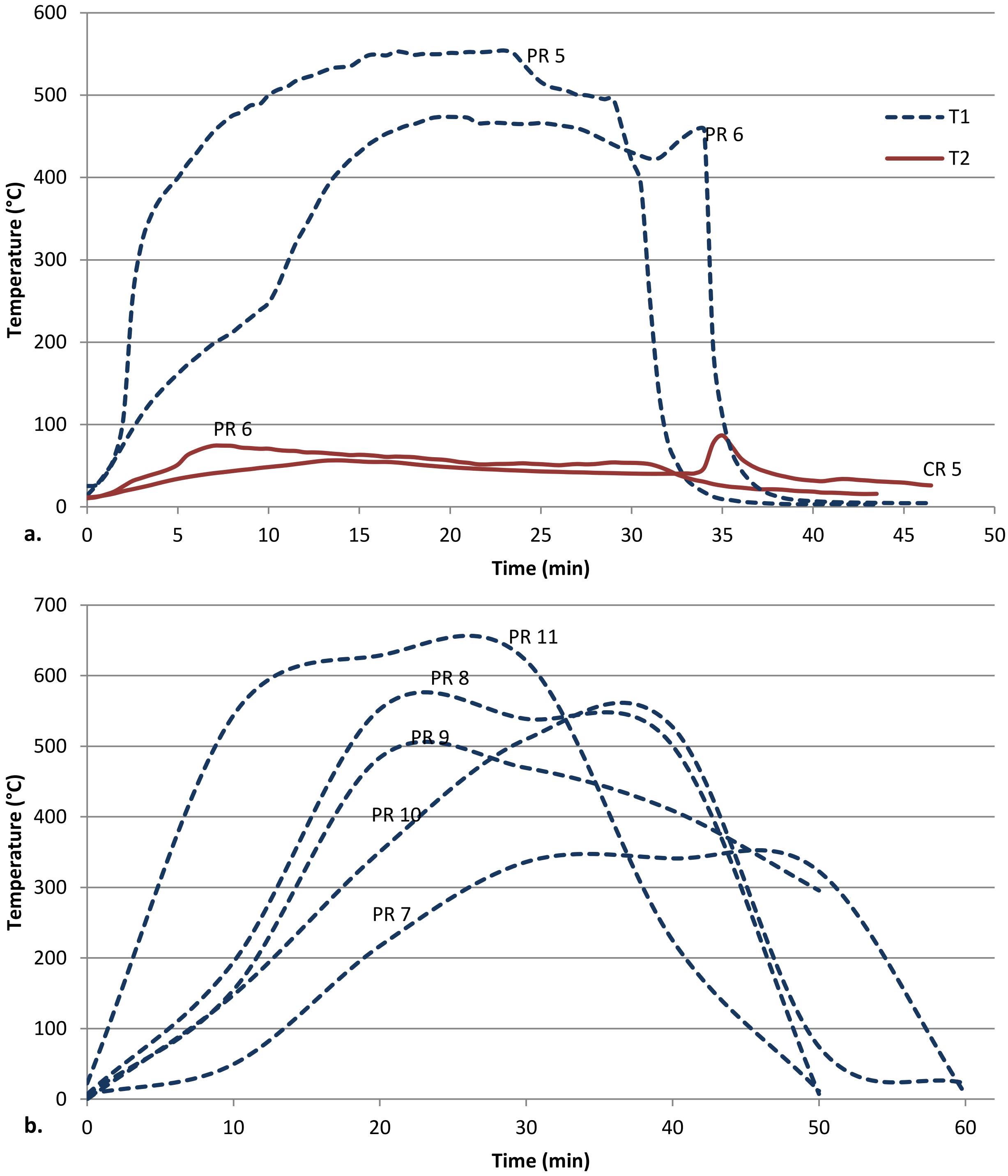

Supplementary Figure S8. Temperature curves for Pit Roll methods PR5, PR6 (a.) and PR7-PR11 (b.). T2 was not recorded for PR7-PR11.Temperatures increase steadily as the embers burn their way through the birch bark, and then decrease as they burn out in the pit. T1 = thermocouple in the middle of the bark roll; T2 = thermocouple in the birch bark vessel in the pit.

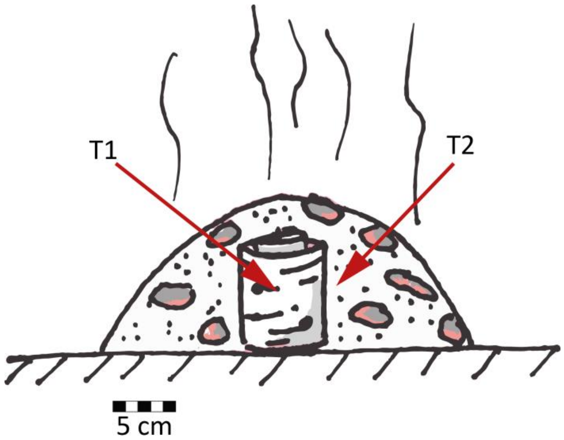

Supplementary Figure S9. Drawing of ash mound method with arrows depicting thermocouple placement. Scale is approximate.

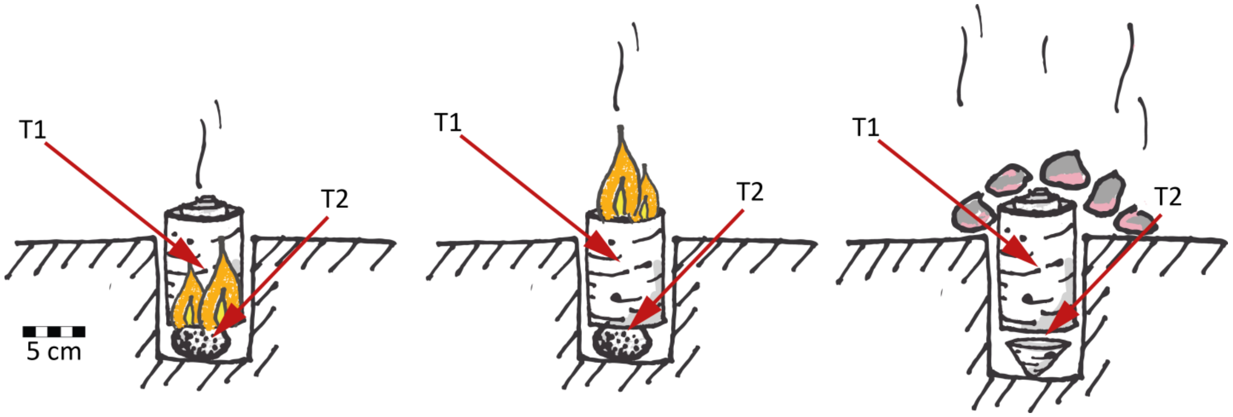

Supplementary Figure S10. Drawing of pit roll methods PR1, PR2 (left); PR3, PR4 (middle), and PR 5-11 (right). Arrows depict thermocouple placement for each method. Scale is approximate.

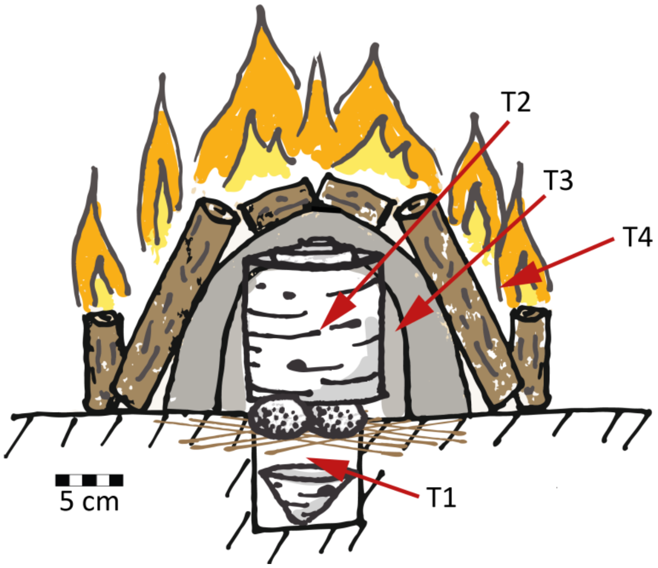

Supplementary Figure S11. Drawing of ash mound method RS7 with arrows showing thermocouple locations. Earlier attempts did not include pebbles under the bark, and used a wooden cup and a metal container as the vessel instead of a birch bark cup shown here. Scale is approximate.

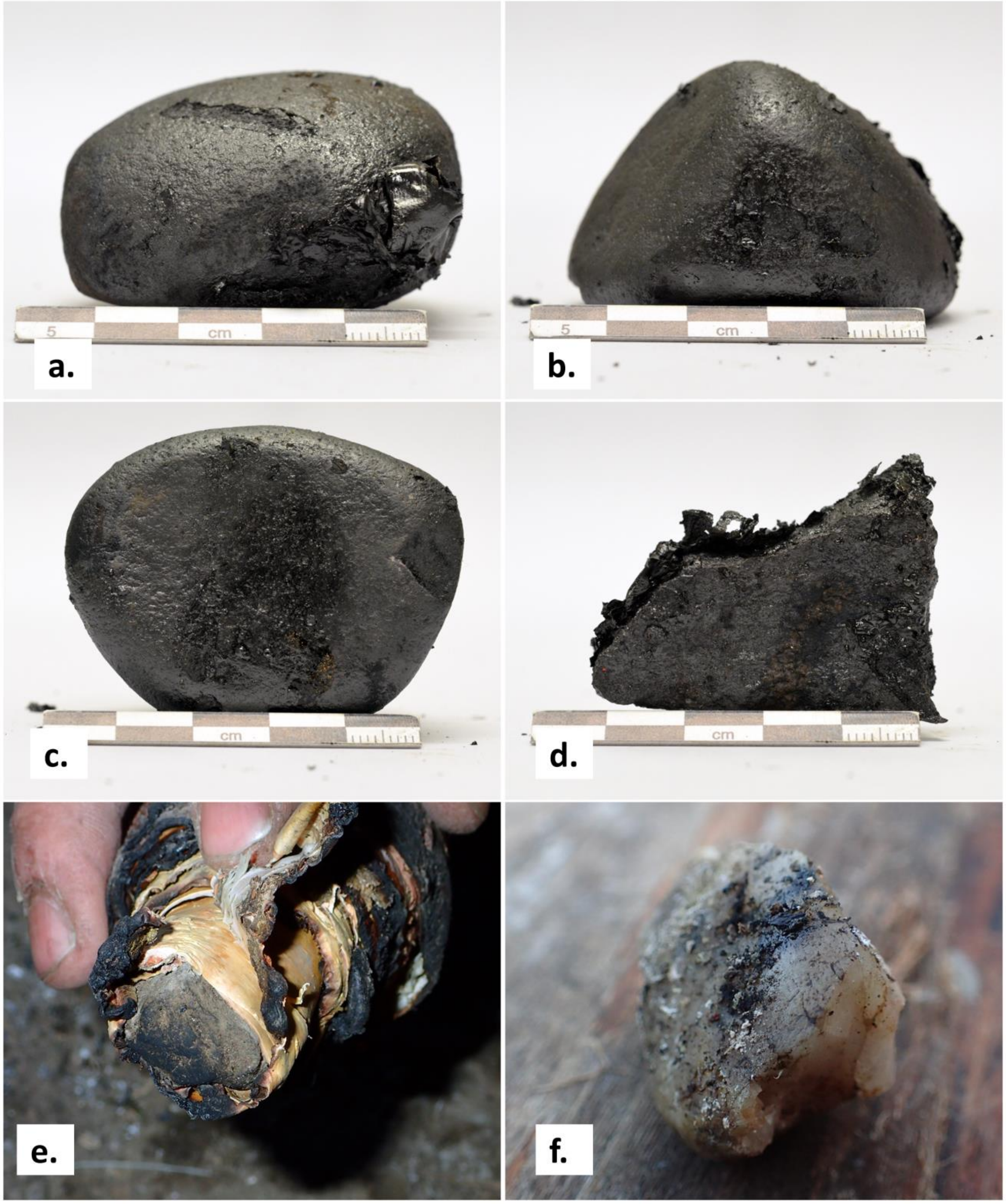

Supplementary Figure S12. Photographs of pebbles used in experiments. a-d) Pebbles used in raised structure; e) and f) pebbles used in pit rolls PR1-PR4. Some charring occurred on the pit roll pebble turning it black, and traces of pyrolysis products appeared on the other side, but only in very small quantities that could not be removed from the pebble or used for hafting in any way.

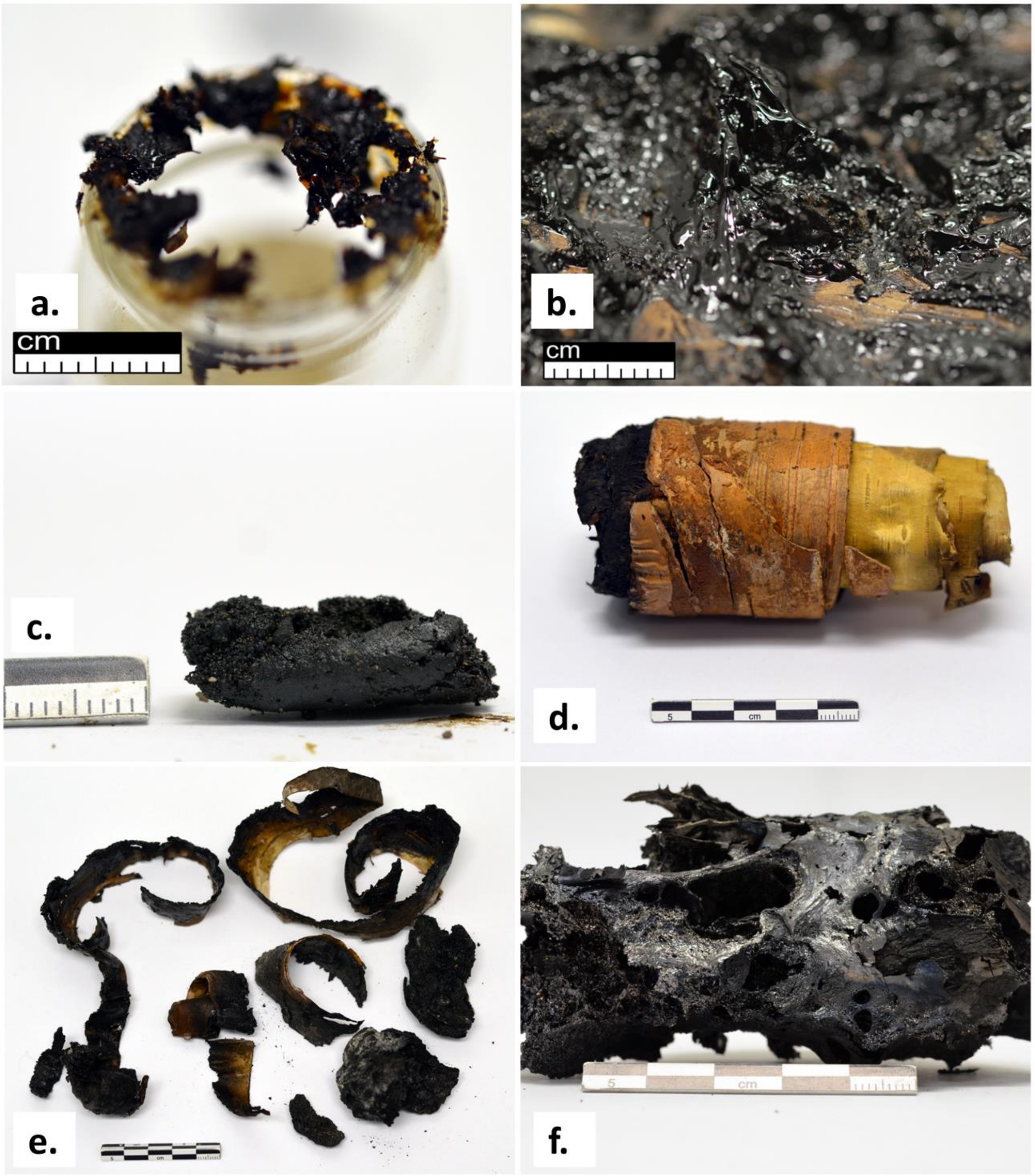

Supplementary Figure S13. Photographs of the remains of various methods and products. a) Some tar from the ash mound method that is highly contaminated with traces of un-charred birch bark; b) tar from the RS7 vessel containing very few contaminants; c) lump of tar from RS8 (using a shell vessel) heavily contaminated in soil. It holds its form, but has very little tack due to the over-saturation of particles; d) remains of bark roll from PR 3 and PR 4. Only slight charring at the end that was ignited and the rest is unchanged; e) remains of bark from successful PR experiment contains much more charred bark; f) bark remains from raised structure RS7. The bark is highly charred and does not resemble birch bark macroscopically.

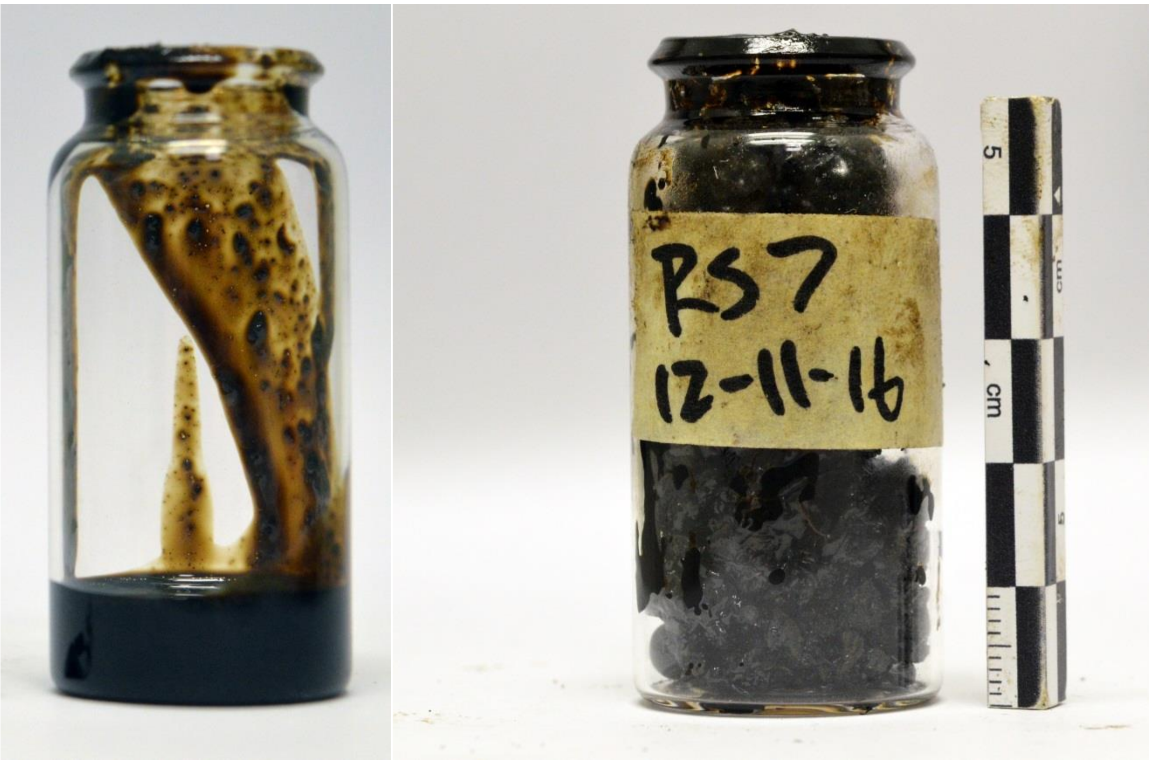

Supplementary Figure S14. Tar from RS 4 (left) and RS 7 (right). RS 4 used a metal container to collect the tar. Temperatures were higher in the metal container (refer above to Fig. S13), but the tar collected remained more liquid than that from RS 7. Suggesting some of the more liquid portion of tar is lost to the earth pit walls, or to the organic vessel.

Notes and comments on aceramic tar production

The following section details our methods, experiences and decisions during the experiments. We included information on: 1) birch bark, 2) fire maintenance, 3) container material and 4) failed attempts and future directions. This information will increase the reproducibility of our work and hopefully spark more much needed research into aceramic tar production.

Bark

Our bark was collected from fresh *Betula pendula* trees, felled the day prior to peeling the bark (August 2016). The bark was used in the experiments five months later (December 2016). Additionally, a small portion (~10%) of bark was collected from trees that had died naturally one year before the experiments. The bark was combined, and selected at random for each experiment. We have seen no evidence to suggest that aged or fresh bark produces more or less tar, but our experiments are unlikely to be consistent enough to see a statistically significant impact regarding the age of the bark.

However, we noticed one difference regarding bark choice between the methods. Thicker and sturdier bark is easier to work with in the ash mount method than thin bark. In this method the bark is unrolled and tar is scraped from the inner surfaces. Thin bark tears easily and it is difficult to unroll thin layers particularly when they are sticky with tar. For the other methods, where tar drips from the bottom of the roll and is collected in a separate container, this is less important, and thinner pieces of bark could be utilised without any detrimental effects. It is currently unknown whether there is a difference in extractive content or quality between thinner and thicker bark remains.

Fire maintenance

For each experimental attempt we had one member conducting the experiment and one or two members recording the information. The data logger recorded temperatures during the experiments, and these were evaluated after each experiment. The temperature and time recordings can be found in Supplementary

Figures S5-S8. Below we detail our (at times subjective) decision making, when determining the best methods.

Ash mound

Prior to beginning tar manufacture with the ash mound, a suitable bed of coals and ash was first produced. The time required for this is available in Supplementary Table S1 under 'fire prep time'. This began at 120 minutes for the first attempt, and we added firewood for between 20 and 40 minutes between each successive ash mound attempt. In this way we could utilize the coals of the previous fire. We maintained a flaming fire until we judged there to be enough ash and embers to cover the small roll of birch bark (approximately 6 cm diameter and 10 cm long). No temperature management was conducted up to this point. When the bark roll was placed in the hearth and surrounded with ash and embers, we judged, based on prior experiences, how hot it should be by moving more or less embers over the bark. When too hot, the bark began to smoke, and black smoke indicated that the bark and tar was burning more completely. As this was undesirable, we would remove some embers and/or add ash. The temperature was judged too low if we did not hear any crackling and did not observe any smoke. In these instances we added glowing embers. We stopped these experiments after approximately 20 minutes; the stop time was relatively constant for the ash mound experiments with the exception of AM3. This decision was based on DP's previous experiences and our own experience with how much bark remained and tar had been produced in the prior experiment. In most cases, although we had to manipulate the ashes and embers to some degree, once we thought there was a good temperature inside the mound, our required attention would decrease, and although we checked it fairly frequently (ever few minutes) we rarely had to change anything else.

Pit roll

We had no previous experience using the pit roll method, other than what had been mentioned in the literature. After trying it as described[1], it was clear that this method would not work. The bark was extinguished very rapidly and no tar was produced. We hypothesised that in order to prevent the bark from

being extinguished we needed to provide an external heat source, as in the ash mound method. To this end, embers were placed on top of the roll of bark and pit. In order to get suitable embers, a flaming fire only needed to be burning for a short time (see Supplementary Table S1). Once these embers were in place, the pit was left alone and required very little further attention, and no further management. However, if the embers were too small to begin with, then the heat provided was not sufficient to produce much tar. The amount of attention required is difficult to quantify objectively, however. We were learning and improving upon this technique and were recording information, thus our attention was relatively high. Had we been more experienced, and not been interested in the experimental aspects, very little attention would be required. Based on our ash mound experience we stopped the experiments after approximately 30-40 minutes. It was also possible to see how much of the bark roll had burned under the embers, and to see when the embers began to die down. As the thermocouple readings corroborate, around this time the temperatures in the pits also began to decline. The pebble in the bottom of the pit showed us that in some cases tar dripped from the bottom of the roll, but this failed to capture anything, so we placed a small container to catch the tar better. In theory this method could be put in place, and left unattended indefinitely. When the operator returns, the embers would have extinguished and the container could be extracted and tar collected. The only downside being that soil contamination is difficult to control in the small pit, and may be worse if the process was left unattended for a longer period.

Raised structure

Regarding the raised structure method, we had previous experience using similar techniques (PK and GL). Prior we used a metal collection vessel under a muddy structure and in another experiment we used metal containers in the 'two-pot-method' (see[2-4] for details of the two-pot and similar methods). From these experiments we knew that the fire needs to run for approximately one hour to dry the 2-3 cm thick muddy structure (longer when the structure is thicker) and one additional hour to make tar. The fires in the experiments here ran for about 2.5-6 hours, depending on how thick or large our structure was.

During the raised structure attempts, the only aim was to maintain a flaming fire all around the structure. When a part of the fire began to burn out, fresh firewood was added to maintain a flame. As the thermocouple was only placed at a single point on this circular fire, temperature logger readings could not help us determine when to place more firewood (for example on the opposite side of the structure). In these experiments the fire was stoked with new wood approximately once every 20-30 minutes, as with a standard campfire, but at the start it was loaded heavily with smaller wood. We perceived this as less demanding than the ash roll method because the temperature of the bark could never be too high (the structure was insulating the bark), and if the fire was diminished it was very simple to replenish it with more wood. Moreover, the span between re-fuelling equalled the total length of the ash mound method, so we could load the fire with wood and then leave it for 20 minutes with minimal attention.

The mound structure itself was made of the earth present in our surroundings with no further alterations. This is a mixture of predominantly clay and earth/soil. We had some problems with the structure cracking, therefore perhaps adding a temper to the clay would help, however it is not completely necessary. Pure sand may be too porous to seal the bark completely, and it may also absorb many of the volatiles that would otherwise condense in the container and form part of the tar. There is likely room for considerable variation in raised structure mound material and consistency. Poor quality material (more porous or more prone to cracking when heated) may need to be thicker, however this will then take longer to heat. Supplementary Figure 6 shows that for RS7 it took approximately 200 minutes to evaporate the moisture from the structure before heating of the internal chamber began. On this attempt we knew we had more bark and a larger structure than before, and thus we left it for as long as possible.

Container material

Despite birch bark being a highly flammable material, we have shown that it survives well in a pit below the fire. This means that a heat resistant retort is not necessary. One important aspect however, is that when only small quantities of tar are produced, the container should be made from non-porous material.

There are many possible containers available that would likely be suitable for collecting tar using these methods, including bone, shell, and eggshell. We used birch bark because it minimises the operational steps of each method, ensuring we did not add unnecessary complexity to the production process.

Failed attempts and future directions

It was clear that the early pit roll methods (PR1-PR4) failed because there was not enough heat for a long enough period of time. With the exception of PR6 (which could not be measured accurately due to high soil contamination), PR11 was the most successful, and also the hottest. This method may possibly be improved by creating a larger roll and/or placing more embers on top.

The first three raised structure attempts were unsuccessful, but we observed that tar was absorbed into our wooden container. Therefore we ran one experiment using a metal container underneath the earthen mound (RS4). This attempt was successful, and showed that we were indeed producing tar, but had previously failed to capture it. For RS5 and RS6 we changed the container to birch bark, which had proved successful in the pit roll methods. Although tar had clearly dripped onto the wooden screen, we still failed to collect a sizable amount. We determined that we needed more bark to ensure that enough tar would be produced to drip through the screen and collect in the vessel. In RS7 we combined our prior experiences and in this successful attempt we produced approximately 16 g tar. During RS7 we also added stones on top of our wood screen. This was done in an attempt to raise the bark higher in the structure (and thus make it hotter), and also to potentially mimic the metal container, which may reflect heat back up, rather than allowing it to dissipate into the cool pit below.

We attempted to duplicate our results in RS8, using a shell container. This method was, however, less successful for a number of reasons. The mud structure appeared to crack more than RS7, and smoke could be seen coming from inside the structure. The smoke indicated that tar volatiles were escaping, and that oxygen may have been present in the structure, leading to combustion rather than distillation. Despite this, tar was still produced and dripped again onto the wood screen, and into the shell. However, the small

and shallow shape of the shell meant it easily filled with soil when we removed the structure. There was also a small crack in the shell, and some tar was found on the underside. All of these aspects combined made it difficult to collect enough tar (likely much of it was disposed of as soil contamination) during this attempt.

There are several potential areas of improvement with the raised structure to ensure a higher success rate, and all of these will lead to increasing complexity. 1) Lining the collection pit with clay or mud to prevent or limit soil contamination during removal. 2) Creating a better mixture for the structure material, such as including more clay, dung, straw etc. to reduce the chances of it cracking when drying. 3) Allowing the structure time to dry before igniting the fire. This would reduce the amount of firewood needed to heat the bark inside the structure. 4) Larger quantities of bark may also help. Perhaps it will not improve the efficiency (tar/bark ratio), but it will ensure enough tar is produced to be captured effectively.

1 Pawlik, A. in *Lithics in Action: Papers from the Conference on Lithic Studies in the Year 2000* (eds E.A. Walker, F. Wenban-Smith, & F. Healy) Ch. 19, 169-179 (Oxbow Books, 2004).

2 Piotrowski, W. in *Experiment and design: Archaeological Studies in Honour of John Coles* Ch. 17, 149-155 (Oxbow Books, 1999).

3 Palmer, F. Die Entstehung von Birkenpech in einer Feuerstelle unter paläolithischen Bedingungen. *Mitteilungen der Gesellschaft für Urgeschichte* **16**, 75-83 (2007).

4 Bacon, G. *Celebrating Birch: The Lore, Art and Craft of an Ancient Tree*. (Fox Chapel Publishing, 2007).
